# Assault Profile and Psychiatric Morbidity in Children With Sexual Abuse: A Community Based Cross- Sectional Study From an Urban Law-Enforcement Centre in India

**DOI:** 10.1192/bjo.2024.258

**Published:** 2024-08-01

**Authors:** Lakshmi Keerthana Thatavarthi, Sireesha S, Aparna M

**Affiliations:** 1 St Johns Medical College and Hospital, Bangalore, India; 2 Osmania Medical College, Hyderabad, India

## Abstract

**Aims:**

The extent and nature of child sexual abuse (CSA) and its consequences with respect to psychiatric morbidity is still poorly described in children. This was a community based cross sectional study to describe the social demographic profile and identify psychiatric morbidity in children with CSA and to further examine the association between the sexual assault profile and the psychiatric illness present.

**Methods:**

This study includes 100 children aged between 6–17 years ascertained as sexually abused at the time. The setting was BHAROSA centre, which is a society for protection of women and at-risk children with funding from the Department of Women and Child Development Telangana state, India. Simple random sampling was used to choose the participants and a pre-tested semi structured questionnaire was used to assess the sexual assault profile. The Developmental Psychopathology Checklist (DPCL) which is the Indian adaptation of Child behaviour checklist was used to understand the associated psychopathology. The prevalence of psychiatric morbidity was discerned by the Diagnostic Statistical Manual Text Revision (DSM V-TR).

**Results:**

The average age for the first CSA encounter was 10.87 years. Most often the perpetrator was found to be an acquaintance (66%) of the child's family. ‘Vaginal/anal penetration’ (55%) was the most common form of abuse. In half of the cases there was a significant delay of two days-two weeks between the last episode of abuse and its discovery. 12% attributed themselves fully responsible for the abuse. 23% reported unsupportive reactions from the caregivers such as being dismissed or being blamed themselves for the abuse. More than half (53%) had at least one psychiatric disorder with post-traumatic stress disorder (PTSD) being the most common (28%) followed by conduct disorder (21%) and depression (17%). 28% had quasi psychotic symptoms and 25% non-specific somatic symptoms. 12% reported suicidal thoughts/ideation. 5 children tested positive for HIV and 2 were pregnant. Children who experienced ‘Vaginal/Anal penetration’ and those who pretended the act did not take place were found to have statistically significant rates of depression, PTSD and suicidality.

**Conclusion:**

All children and adolescents who have been sexually abused must be evaluated for psychiatric morbidity regardless of their social demographic and abuse profiles. Additionally, all parents and caregivers should be sensitised on the fact that the majority of the perpetrators are acquaintances to the subjects. Coping strategies of the children especially self-blame and poor social support exert direct negative effects on victims’ adjustment.

